# Prevalence and predictors of metabolic abnormalities in Chinese women with PCOS: a cross- sectional study

**DOI:** 10.1186/1472-6823-14-76

**Published:** 2014-09-16

**Authors:** Rong Li, Geng Yu, Dongzi Yang, Shangwei Li, Shulan Lu, Xiaoke Wu, Zhaolian Wei, Xueru Song, Xiuxia Wang, Shuxin Fu, Jie Qiao

**Affiliations:** 1 Department of Obstetrics and Gynecology, Reproductive Medical Center, Peking University Third Hospital, No. 49 North Huayuan Road, Haidian District, Beijing 100191, China; 2 Department of Obstetrics and Gynecology, Sun Yat-Sen Memorial Hospital, Sun Yat-Sen University, Guangdong, China; 3 Department of Obstetrics and Gynecology, Reproductive Medical Center, West China Second University Hospital, Sichuan University, Chengdu, China; 4 Department of Obstetrics and Gynecology, First Affiliated Hospital of Medical College of Xi’an Jiaotong University, Xi’an, China; 5 Department of Obstetrics and Gyneocology, First Affiliated Hospital of Heilongjiang Chinese Medicine University, Harbin, China; 6 Department of Obstetrics and Gynecology, First Affiliated Hospital of Anhui Medical University, Anhui, China; 7 Department of Obstetrics and Gynecology, Tianjin Medical University General Hospital, Heping, China; 8 Department of Obstetrics and Gynecology, Shengjing Hospital of China Medical University, Shenyang, China; 9 Department of Obstetrics and Gynecology, Second Xiangya Hospital of Central-South University, Changsha, China

**Keywords:** Prevalence, Predictor, Metabolic abnormalities, PCOS, Community

## Abstract

**Background:**

Polycystic ovary syndrome (PCOS) is a common condition estimated to affect 5.61% of Chinese women of reproductive age, but little is known about the prevalence and predictors in Chinese PCOS patients. This study aimed to determine the prevalence and predictors of the metabolic abnormalities in Chinese women with and without PCOS.

**Methods:**

A large-scale national epidemiological investigation was conducted in reproductive age women (19 to 45 years) across China. 833 reproductive aged PCOS women, who participated in the healthcare screening, were recruited from ten provinces in China. Clinical history, ultrasonographic exam (ovarian follicle), hormonal and metabolic parameters were the main outcome measures.

**Results:**

The prevalence of metabolic syndrome (MetS) as compared in PCOS and non-PCOS women from community were 18.2% vs 14.7%, and IR (insulin resistance) were 14.2% vs 9.3% (p < 0.001) respectively. After adjusting for age, the indicators (central obesity, hypertension, fasting insulin, SHBG, dyslipinaemia) for metabolic disturbances were significantly higher in PCOS than in non-PCOS groups. Using multivariate logistic regression, central obesity and FAI were risk factors, while SHBG was a protective factor on the occurrence of Mets and IR in PCOS women (OR: 1.132, 1.105 and 0.995).

**Conclusions:**

The risk factors of the metabolic syndrome and insulin resistance were BMI and FAI for PCOS women, respectively. The decrease of SHBG level was also a risk factor for insulin resistance in both PCOS and metabolic disturbance.

## Background

Polycystic ovary syndrome (PCOS) is a common condition estimated to affect 5.6% of Chinese women of reproductive age [1,2]. PCOS is associated with reproductive and metabolic disturbances [3]. Based on the Adult Treatment Panel III criteria [4], the prevalence of metabolic syndrome (MetS) has been previously reported to be 1.6% [5], 8.2% [6] and 43% [7] in Czech, Italian and US women with PCOS, and 24.9% in Chinese Hongkong women [8]. The prevalence of MetS in PCOS women showed a marked variation between countries and ethnic groups, probably due to differences in diet, lifestyle and genetic factors. In addition, it was also associated with the investigated population. A meta-analysis supported a greater prevalence of glucose intolerance (IGT), Types 2 diabetes (DM2) and the metabolic syndrome in women with PCOS as compared with women without PCOS [9]. The odds of metabolic disturbance were two to four times as high in PCOS women [9]. The predisposition of PCOS women to various metabolic disturbances, including obesity, IGT, atherogenic dyslipidaemia and hypertension, increased in the long-term risk of DM2 and cardiovascular disease (CVD), which indicated that PCOS carried significant public health implication [10]. Recent evidence also indicated more frequent CVD death in women with PCOS [10]. An economic evaluation estimated that 40% of the economic costs of PCOS can be attributed to DM2 in the USA, highlighting the need for prevention of long term complications through appropriate screening, diagnosis and intervention for PCOS [10].

Insulin resistance (IR) is the most likely the pathogenic link between PCOS and MetS. The co-morbidities associated with IR are common to both conditions. All surrogate markers of reduced insulin sensitivity have consistently been found in women with concomitant PCOS and MetS compared to those without MetS, even after controlling for Body Mass Index (BMI) [11]. Some evidence suggested that women with PCOS had a greater predisposition to obesity.

The increased risk of MetS and IR in women with PCOS has raised further interest in identifying the predictors for MetS and IR in these women. The objective of this study was to investigate the prevalence of the metabolic syndrome in PCOS women in China, and detect the predictive risk factors in metabolic disturbances in order to find an effective tool to screen for potential CVD risk factor in Chinese women with PCOS.

## Methods

**Figure 1:**
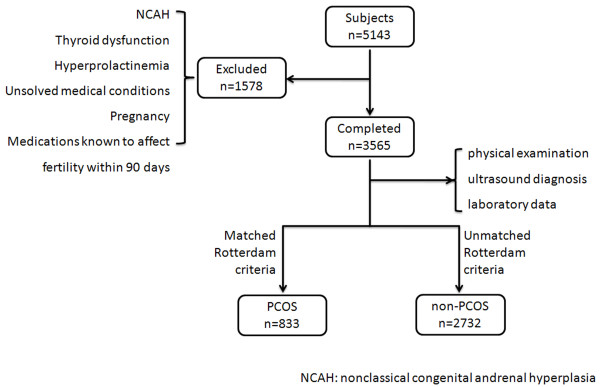
Route diagram of the present prevalence study.

Anthropometric and metabolic measures were performed for women who participated in the epidemiological study of PCOS around China. All participants were systematically evaluated and written informed consent was obtained from all participants. The study route was in the flowchart, Figure 1.

The diagnosis of PCOS was based on Rotterdam-PCOS criteria [12]. According to these criteria, PCOS were diagnosed if at least two of the following criteria were present: oligo/amenorrhoea, clinical or biochemical hyperandrogenism and PCO on ultrasonography. Other etiology that could mimic PCOS, like Cushing syndrome, late onset adrenal hyperplasia or androgen producing neoplasm had to be excluded.

Oligomenorrhea and amenorrhea were defined as having fewer than 8 menstrual cycles per year, or the absence of 3 to 6 consecutive menstrual cycles per year.

Clinical hyperandrogenism was defined as the presence of hirsutism (Ferriman-Galwey score ≥6, no oral contraceptive pills were used within three months). Biochemical hyperandrogenism was present if testosterone >2.8 nmol/L or androgen > 10.8 nmol/L, which were the normal range of 95% percentile in the population in our laboratory. PCO was defined as the presence of at least one ovary with 12 or more follicles measuring 2-9 mm in diameter.

MetS was defined according to modified NCEP ATP III guidelines 2005 [13]. MetS was diagnosed if at least three of the following five measures were present: (i) waist circumference ≥ 80 cm, (ii)serumtriglyceride ≥ 1.7 mmol/l, (iii) serum high-density lipoprotein (HDL)-cholesterol < 1.3 mmol/l or the use of lipid lowering medication, (iv) blood pressure ≥130/85 mmHg or the use of anti-hypertensive (v) fasting plasma glucose ≥5.6 mmol/l.

IR was evaluated by using the homeostatic model assessment (HOMA-IR: (fasting insulin × fasting glucose)/22.5) and 2.69 was selected as the cut-off point [14].

A large-scale national epidemiological investigation was conducted in women of reproductive age (19 to 45 years) from the top ten provinces and municipalities in China [1]. Ten geographically distributed provinces/municipalities contained the major residential population from 30 provinces of the whole China. All selected participants were chosen from both rural and urban communities (1:1). Ten provinces were chosen from all 30 provinces according to geographically distributed, then used a multi-layer, stratified sampling method from city or district to communities. A total of 5163 women underwent the investigation from 2008 to 2009. Only 3565 participants completed the panel of physical examination, ultrasound and laboratory data for the classification of PCOS. Twenty interviewers, gynaecologists and ultrasonographcis were trained to administrate the standardized questionnaire and conduct, physical examination and ultrasound following the same Standard Operating Procedure. MetS and IR, the main issue, were considered for analysis. Other disorders such as nonclassical congenital and renal hyperplasia, thyroid dysfunction, and hyperprolactinemia were excluded by examining medical histories and hormones tests, and we deemed the abnormality for prolactin (PRL ≥25 ng/ml) and thyroid dysfunction (TSH <0.5 mU/Lor >4.78 mU/L), according to the reference value. Other exclusionary criteria included unsolved medical conditions, pregnancy and the use of medications known or suspected to affect fertility or metabolic function with 90 days of the study entry was prohibited. This study was approved by the Ethics Committee of Peking University Third Hospital and conducted based on guidelines of the institutional review boards in each of the participating centres. All subjects provided written informed consent.

### Physical examination, ultrasound and laboratory assessment

Blood pressure was obtained in sitting patients after a five-minute rest. Waist circumference was measured in the standing position, halfway between the lower ribs and the superior anterior iliac spine of the pelvic. The hip circumference was measured at the level of the pubic symphysis. Hirsutism was established by using the modified Ferriman-Galwey score [15]. Transvaginal ultrasonography was performed by the investigators to detect PCO. Height and body weight were measured, and BMI value were calculated. The BMI values were classified following the established criterion, that is normal (23 kg/m^2^ ≤ BMI < 25 kg/m^2^), overweight (25 kg/m^2^ ≤ BMI 30 < kg/m^2^) and obese (BMI ≥ 30 kg/m^2^) obese in Asian [16].

### Laboratory tests

All blood samples were collected in the morning after fasting for at least 8 hours. Fasting plasma glucose (FPG) was measured by using finger stick blood glucose method (Roche ACCU-CHEK). Venous blood sample were collected and were immediately centrifuged, then the serum was separated and frozen at -20°C until assayed. All blood samples’ test was done in Peking University Third Hospital. Fasting insulin (FI), SHBG, total testosterone (TT), androstenedione (A), PRL and TSH were assessed by chemiluminescence under the Immulite 1000 (DPC, USA). Manufacturer’s instructions were followed for preparation, set-up, dilutions, adjustments, assay, and quality control procedures. For all measurements, the inter-assay coefficient of variation was less than 10%, while the intra-assay variation was less than 15%. Fasting cholesterol, triglyceride (TG), low-density lipoprotein (LDL) and high-density lipoprotein (HDL) were measured by dry slide enzymatic colorimetric assay. The free androgen index (FAI) was calculated using the formula [TT (nmol/L) × 100/SHBG (nmol/L)].

### Statistical analysis

All analyses were performed by using Statistical Product and Service Solutions (SPSS) (version 13.0, spss inc., Chicago, IL, USA). Continuous variables were presented as mean ± SD or median (interquartile range), and analysed using independent sample t-test for normally distributed data or Mann–Whitney *U*-test for skewed data. Categorical variables were expressed as proportion (percentage) and analysed by *X*^2^ or Fisher’s exact tests as appropriate. Multivariate logistic regression was used to examine independent predictors of MetS and IR. The results were expressed as age and BMI adjusted odds ratio (OR) with 95% confidence interval (CI) or two sided p-value. Baseline characteristics of the different PCOS phenotype and controls were evaluated by analysis of variance. Proportion was compared using the chi-square test. Univariate logistic regression analysis was applied to qualify the association between several clinical and laboratory variables, and the presence of the metabolic disturbances. Variables that appear to be associated were further analysed using multivariate logistic regression analysis with backwards stepwise selection. Statistical significance was considered present if the P-value was <0.05.

## Results

### Prevalence of the metabolic syndrome and insulin resistance in different phenotype PCOS in community

The prevalence of the metabolic syndrome and insulin resistance were 19.1% (159/833) and 14.2% (118/833) in women with PCOS. Table 1 showed the prevalence of MetS in different PCOS phenotype subgroups was similar (Table 1).

**Table 1:** Prevalence of MetS and IR in different phenotypes in PCOS women

	**IM + HA + PCOM**	**IM + HA**	**HA + PCOM**	**IM + PCOM**	**P-value**
Number in community	28.69% (239/833)	18.97% (158/833)	37.33% (311/833)	15.01% (125/833)	<0.001
MetS(ATPIII2005) in community	20.5% (49/239)	17.1% (27/158)	18.6% (58/311)	20.0% (25/125)	0.87
IR in community	16.7% (40/239)	13.9% (22/158)	12.9% (40/311)	12.8% (16/125)	0.20

IM: irregular menstruation; HA:hyperandrogenism; PCOM: pco morpholity.

### Clinical and biochemical characteristics of the PCOS and non-PCOS

Of the 3,565 women screened, 833 were diagnosed as PCOS according to Rotterdam-PCOS criteria, and 2,732 were non-PCOS population. Compared women without PCOS, those with PCOS were younger, but there was no difference in central obesity as measured by Waist to hip ratio (WHR). However, since age and central obesity were closely correlated, the indicators for central obesity in PCOS women were significantly higher than in non-PCOS when adjusted for ages.

In addition, T, A, FAI were significantly higher, while SHBG was lower in PCOS than non-PCOS women. After controlling for age and BMI, that was no difference in blood pressure, cholesterol, HDL, LDL, FPG, FI, HOMA-IR between the two groups, with the exclusion of TG which was higher in PCOS.

### Prevalence of the metabolic syndrome and insulin resistance in PCOS and non-PCOS women

The prevalence of MetS (ATPIII2005) was 19.1% (159/833) and 14.7% (401/2732) (P < 0.001) respectively, in PCOS women and non-PCOS women. In addition, 34.1, 18.6, 13.0, 4.8, 1.3% of women with PCOS had at least one, two, three, four or five components of Mets (ATPIII2005), respectively. The frequency of each component of metabolic syndrome population, in decreasing order, was reduced HDL-C (85.9%), central obesity (84.8%), increased TG (63.4%), increased fasting glucose (55%), and hypertension (45.7%).

**Figure 2:**
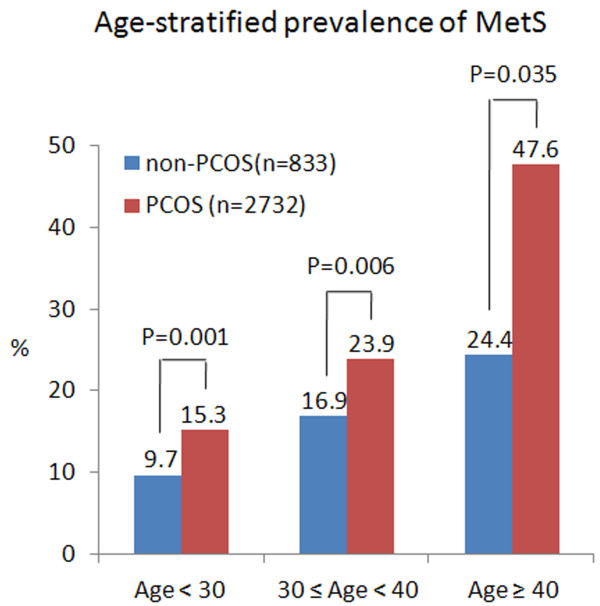
**Age-stratified prevalence of MetS in PCOS.** Diagnosis of MetS based on the modified Adult Treatment Penal III (ATP2005).

**Figure 3:**
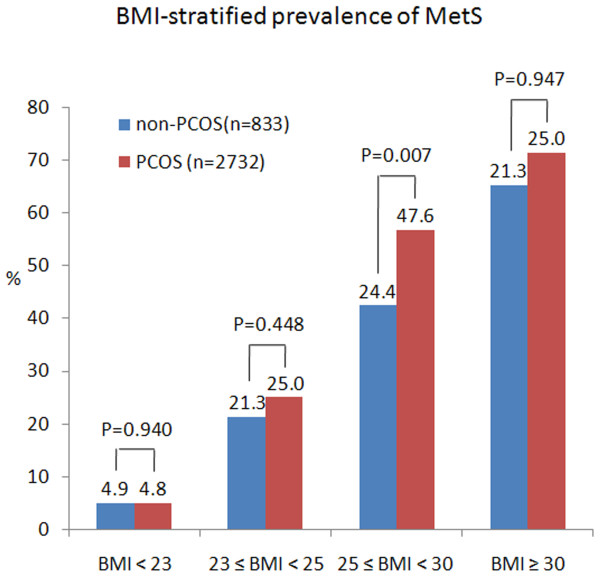
**BMI-stratified prevalence of MetS in PCOS.** Diagnosis of MetS based on the modified Adult Treatment Penal III (ATP2005).

The prevalence of IR was 14.2% (118/833) and 9.3% (254/2732) respectively, with P <0.001. After adjusted for age and BMI, the prevalence of IR and MetS between the two groups was still significantly different (Table 2).We performed stratified analysis, by age and weight category, since age and BMI were well known confounding factors for MetS (Figure 2 and Figure 3). As was expected, the prevalence of MetS increased with increasing age and was significantly higher in PCOS than non-PCOS women in all age groups. IR was also significantly higher with age in PCOS. When compared the prevalence of MetS after stratification into subgroups of normal-weight or overweight/obese, we found the prevalence of MetS was dramatically increased with increasing BMI, and obese II group had 13–16 fold risk of MetS than normal weight woman, irrespective of the presence of PCOS. In the four groups, the prevalent differences of PCOS and non-PCOS women were not significant except obese I group (p = 0.006) (Figure 3).

**Table 2:** Comparison of women with and without PCOS

	**PCOS**	**Non-PCOS**	**P-value**	**Age adjusted P- value**	**Age and BMI adjusted p-value**
No. of subject	833	2,732			
Age	29.1 ± 5.4	32.3 ± 6.1	<0.001		
BMI	22.3 ± 3.9	22.2 ± 3.2	0.33	<0.001	
Weight	56.8 ± 10.7	56.1 ± 8.8	0.09	<0.001	0.03
Height	159.4 ± 5.1	159.0 ± 5.5	0.04	0.04	0.03
Waistline	75.9 ± 10.5	75.6 ± 9.1	0.37	<0.001	0.24
WHR	0.83 ± 0.07	0.83 ± 0.07	0.59	<0.001	0.08
SBP	111.3 ± 13.9	111.6 ± 13.8	0.57	0.08	0.82
DBP	73.2 ± 10.2	73.1 ± 9.8	0.92	0.01	0.99
FPG	5.2 ± 1.0	5.1 ± 1.0	0.04	0.11	0.21
FI	4.0 (2.0-7.4)	3.4 (2.0-6.0)	<0.001	0.04	0.67
HOMA-IR	0.9 (0.5-1.7)	0.8 (0.5-1.4)	<0.001	0.09	0.50
Cholestrol	4.5 ± 1.0	4.5 ± 1.0	0.21	0.04	0.12
HDL	1.4 ± 0.4	1.4 ± 0.3	0.08	0.01	0.10
LDL	2.3 ± 0.7	2.3 ± 0.7	0.29	0.13	0.39
SHBG	50.6 (34.5-70.9)	59.4 (42.3-81.0)	<0.001	<0.001	<0.001
FAI	3.7 (2.3-6.1)	2.1 (1.4-3.4)	<0.001	<0.001	<0.001
TG	1.0 (0.7-1.5)	1.0 (0.7-1.4)	0.83	<0.001	0.03
T	1.9 (1.4-2.5)	1.3 (0.8-1.8)	<0.001	<0.001	<0.001
A	12.5 (10.2-15.2)	8.3 (6.2-10.5)	<0.001	<0.001	<0.001

Comments: Data were presented as means ± SD, proportion (%) or median (inter-quartile range), and analysed by independent sample t-test, X^2^/fisher’s exact tests, or Mann–Whitney U-test as appropriated. BP, blood pressure; TG, triglycerides; HDL-C, high-density lipoprotein cholesterol; LDL-C, low-density lipopreotein cholesterol; FPG, fasting plasma glucose; FI, fasting insulin.

### Characteristics of PCOS women with and without metabolic syndrome

As shown in Table 3, women with the metabolic syndrome were older and more obese. They also had lower SHBG concentrations, higher FAI and reduced insulin sensitivity as manifested by greater HOMA-IR and higher fasting insulin concentrations than those without MetS. There were no significant differences in serum total testosterone or androgen between those PCOS women with and without MetS. The proportion of HA, IM, PCOM were not significantly different between PCOS woman with MetS and without MetS.

**Table 3:** Comparison of PCOS women with MetS and without MetS

	**Non-Mets2005**	**MetS2005**	**P-value**	**Age and BMI adjusted p-value**
No. of subject	674	159		
Age	28.7 ± 5.3	30.1 ± 5.5	<0.001	
BMI	21.5 ± 2.8	25.9 ± 3.7	<0.001	
Weight	54.0 ± 8.4	68.3 ± 11.7	<0.001	0.44
Height	159.4 ± 5.0	159.7 ± 5.6	0.49	0.03
Waistline	73.0 ± 8.3	88.0 ± 10.2	<0.001	<0.001
WHR	0.81 ± 0.06	0.89 ± 0.06	<0.001	<0.001
SBP	108.5 ± 11.3	123.2 ± 17.0	<0.001	<0.001
DBP	71.1 ± 8.7	80.8 ± 11.7	<0.001	<0.001
FG	5.1 ± 0.9	5.6 ± 1.2	<0.001	<0.001
FI	3.5 (2.0-5.9)	8.6 (4.7-14.2)	<0.001	<0.001
HOMA-IR	0.8 (0.5-1.3)	2.2 (1.2-3.7)	<0.001	<0.001
Cholesterol	4.5 ± 1.0	4.6 ± 0.9	0.36	0.17
HDL	1.4 ± 0.3	1.1 ± 0.2	<0.001	<0.001
LDL	2.3 ± 0.8	2.4 ± 0.7	0.09	0.47
SHBG	55.4 (40.6-75.6)	27.9 (19.8-45.5)	<0.001	<0.001
FAI	3.3 (2.1-5.2)	6.4 (3.4-10.2)	<0.001	<0.001
TG	0.9 (0.7-1.2)	2.0 (1.3-2.5)	<0.001	<0.001
T	1.9 (1.4-2.6)	1.9 (1.3-2.5)	0.39	0.13
A	12.4 (10.2-15.3)	12.7 (10.4-14.9)	0.94	0.56
IM	62.5% (421/674)	63.5% (101/159)	0.44	0.43
HA	85.2% (574/674)	84.3% (134/159)	0.43	0.76
PCOM	80.6% (543/674)	83.0% (132/159)	0.28	0.23

Comments: Data were presented as means ± SD, proportion(%) or median(inter-quartile range), and analysed by independent sample t-test, X^2^/fisher’s exact tests, or Mann–Whitney U-test as appropriated. BP, blood pressure; TG, triglycerides; HDL-C, high-density lipoprotein cholesterol; LDL-C low-density lipopreotein cholestrol. FPG, fasting plasma glucose; FI, fasting insulin: IM: irregular menstruation HA:hyperandrogenism PCOM: pco morpholity.

### Predictors of MetS and IR by using multivariate logistic regression analysis

Multivariate logistic regression was also performed to examine the independent predictors of Mets and IR in all women with and without PCOS. For women with PCOS, BMI (OR: 1.420, 95% CI 1.328-1.518) and FAI (OR: 1.132, 95% CI 1.037-1.236) were risk factors for MetS, while SHBG (OR: 0.995, 95% CI 0.990-1.000) was protective factor. For women without PCOS, the risk factor was BMI (OR: 1.381, 95% CI 1.324-1.440).

Predictive factors for the presence of IR in women with PCOS were BMI (OR: 1.211, 95% CI 1.147-1.279), A (OR: 1.067 95% CI 1.022-1.114) and FAI (OR: 1.105, 95% CI 1.049-1.165), while the protective factor was SHBG (OR: 0.985, 95% CI 0.976-0.994). For women without PCOS, predictive factors were BMI (OR: 1.186, 95% CI 1.140-1.234), and FAI (OR: 1.120, 95% CI 1.053-1.193) (Table 4).

**Table 4:** Predictors of MetS and IR by using multivariate logistic regression analysis

		**OR**	**95.0% CI**	**P**
PCOS/ MetS	BMI	1.420	1.328-1.518	0.000
	FAI	1.132	1.037-1.236	0.006
	SHBG	0.995	0.990-1.000	0.086
Non-PCOS/ MetS	BMI	1.381	1.324-1.440	0.000
PCOS/ IR	BMI	1.211	1.147-1.279	0.000
	A	1.067	1.022-1.114	0.011
	FAI	1.105	1.049-1.165	0.000
Non-PCOS/IR	BMI	1.186	1.140-1.234	0.000
	FAI	1.120	1.053-1.193	0.000

## Discussion

In the present study, a large-scale national epidemiological investigation was conducted in reproductive age women (19 to 45 years) across China, and total 833 reproductive aged PCOS women who were accordance with the established criterions participated from ten provinces in China. Compared with non-PCOS patients, we found that obesity and FAI were both risk factors for PCOS women, while SHBG acted as a protective factor role on the occurrence of Mets and IR in PCOS women.

### Prevalence of metabolic disturbances in PCOS and non-PCOS women

The consequences of the polycystic ovary syndrome extended beyond the reproductive axis; as women with PCOS are at substantial risk for the metabolic syndrome. Also, previous studies indicated that 30–40 percent women with PCOS have impaired glucose tolerance, and as many as 10 percent have type 2 diabetes by their fourth decade [17,18].

In agreement with the previous studies, we demonstrated that a higher prevalence of MetS (ATPIII2005) and IR in women with PCOS than those without PCOS. After controlling for the age and BMI, the difference was still significant, indicating PCOS women had a higher risk of metabolic disturbance compared to the controls.

The prevalence of MetS and IR were significantly higher in PCOS women than non-PCOS women in general population of China. The PCOS phenotype had no effect on the occurrence of MetS. However, previous study concluded that obesity was strongly associated with MetS in adolescent women, whether they had PCOS or not [19]. Obesity appeared to be closely associated with PCOS. For example, in the United States, more than half of the patients with PCOS are either overweight or obese [20]. Our data also showed the PCOS women are more obese than control, but the percentage of overweight/obesity was not significantly different between PCOS (33.9%) women and Non-PCOS women (34.4%). Our study demonstrated that the metabolic disturbance was occurred more often in overweight and obese women. It was interesting that SHBG level was significantly lower in PCOS women compared with non-PCOS women, as well as SHBG level was also significantly lower in MetS than in non-MetS. Since the SHBG had correlation to the IR [21]. This study indicated that SHBG was a well- established marker of insulin resistance in diabetics [22], and low levels had been reported in adolescent girls with premature pubarche (who were known to be at risk for PCOS and insulin resistance) [23]. Our results regarding the SHBG were protective factor, which was not reported by other research. We interpreted that the variation in SHBG levels might mostly result from the effects of IR/hyperinsulinemia upon hepatic SHBG production. Thus a low level of SHBG might serve as another marker of insulin resistance, and a key factor during the pathogenesis of both PCOS and MetS.

### Relationship of hyperandroginaemia and metabolic abnormalities in PCOS

The relationship between hyperandrogenaemia and metabolic abnormalities is controversial. Apridonidze et al. described a higher prevalence of hyperandrogenaemia in women with concomitant PCOS and MetS [7], others study concluded that DHEAS correlated inversely with arterial structure, suggesting possible cardio-protective effects of endogenous DHEAS in women with PCOS [24]. However, our data, similar studies from Dokras et al. [11] and L.P. Cheung et al. [8], all failed to demonstrate any significant differences in serum concentrations in total testosterone and androgen between those PCOS women with or without MetS. Therefore, it appeared that hyperandrogenaemia, by itself, may not directly contribute to the development of MetS in women with PCOS.

Considering the higher prevalence of insulin resistance in the PCOS women, we analyzed the high risk factors including BMI, testosterone, androgen, SHBG and FAI as the predictors of IR in PCOS women.

Currently, it was shown that lipid abnormalities and hyperinsulinaemia persisted despite suppression of androgens with GnRH agonists in hirsute hyperandrogenic women [25]. The recognition of the role of insulin resistance, rather than hyperandrogenism, as the main culprit in the pathogenesis of MetS in PCOS had an important therapeutic implication. Due to the higher prevalence of MetS and IR in the PCOS women, and serious complications such as CVD and DM2, it was also important to treat these diseases beyond the fertility aspects.

The strength of the study was the large scale investigation, and all the women participated were recruited from the general population. AS compared with other studies on MetS in PCOS, most PCOS women were recruited from the hospital or clinics, which might contribute to potential selection bias and over estimated the risk of MetS and IR in the PCOS cohort. However, there were also several limitations of this study. We measured total testosterone concentration and FAI, which might be a less sensitive marker for hyperandrogenism than free testosterone levels. . In the present study, being the first large scale study in general population, might provide valuable insight towards better understanding of IR and MetS profiles in PCOS women in China.

## Conclusion

In conclusion, the prevalence of MetS and IR were significantly higher in PCOS women than non-PCOS women in Chinese community, and their risk factors were BMI and SHBG. Moreover FAI was also one of risk effects on the insulin resistance in PCOS women since the hyperandroginaemia. The present study facilitated the understand for PCOS pathological characteristics, and was helpful to seek the new target for treatment.

## Abbreviations

PCOS: Polycystic ovary syndrome; MetS: Metabolic syndrome; IGT: Glucose intolerance; DM2: Types 2 diabetes; CVD: Cardiovascular disease; IR: Insulin resistance; BMI: Body Mass Index; SHBG: Sex Hormone Binding Globulin; TSH: Thyroid Stimulating Hormone; HOMA: Homeostatic model assessment; FPG: Fasting plasma glucose; FI: Fasting insulin; TT: Total testosterone; A: Androstenedione; HDL: High-density lipoprotein; TG: Triglyceride; LDL: Low-density lipoprotein; OR: Odds ratio; WHR: Waist to hip ratio.

## Competing interests

The authors declare that they have no competing interests.

## Authors’ contributions

RL contributed to the acquisition of Beijing and Henan provinces’ data, drafting the article and revising it critically for important intellectual content. GY was mainly in charge of the data analysis and drafting the article. DY, SL, SL, XW, ZW, XS, XW and SF did the specific data interpretation of Guangdong, Sichuan, Shanxi, Heilongjiang, Anhui, Tianjin and Hunan province, respectively. JQ contributed to conception and design, final approval of the version to be published as corresponding author. All authors read and approved the final manuscript.

## Pre-publication history

The pre-publication history for this paper can be accessed here:

http://www.biomedcentral.com/1472-6823/14/76/prepub
